# Developing a Practical Welfare Assessment Tool for Intensive Sheep and Goat Farming in Hot-Arid Regions: Pilot Validation in the United Arab Emirates

**DOI:** 10.3390/ani16040563

**Published:** 2026-02-11

**Authors:** Ebru Emsen, Muzeyyen Kutluca Korkmaz, Bahadir Odevci, Aysha Alnuaimi, Maryam Almarzooqi, Anoud Alketbi, Dana Alhammadi

**Affiliations:** 1 Integrative Agriculture, College of Agriculture & Veterinary Medicine, United Arab Emirates University, Al Ain P.O. Box 15551, United Arab Emirates; 2 Department of Animal Science, Faculty of Agriculture, Malatya Turgut Ozal University, Malatya 44210, Turkey; muzeyyen.korkmaz@ozal.edu.tr; 3 Imona Technologies, ITU Ari Teknokent, Istanbul 34485, Turkey; bahadir.odevci@imona.com

**Keywords:** animal welfare, sheep, goats, hot-arid climate, welfare indicators, inter-observer reliability, welfare index, intensive farming

## Abstract

Intensive sheep and goat farming in hot-arid regions faces unique welfare challenges that differ substantially from those encountered in cooler climates; however, few practical and validated assessment tools are specifically designed to assess welfare under such extreme conditions. In this study, the term practical refers to field feasibility under routine farm conditions, limited assessment time, and suitability for reliability-based application, rather than comprehensive validation of welfare outcomes. This study aimed to develop and pilot-test a simplified welfare assessment protocol, based on a reduced set of clearly defined, field-applicable indicators supported by explicit operational definitions and standardized scoring criteria, tailored for the United Arab Emirates, with a specific focus on extreme heat and intensive husbandry conditions. Candidate indicators were identified from validated international sources and screened for applicability to arid climates, meat-oriented production, and intensive systems. The refined indicator set was converted into operational scoring sheets and applied by trained undergraduate animal science students as assessors to 100 animals at an intensive research farm. Inter-observer reliability was calculated using Fleiss’ Kappa to evaluate consistency across assessors. Most behavioural and health indicators demonstrated substantial to almost perfect inter-observer agreement (κ-based), while environmental and some tactile indicators, such as body condition and hydration tests, showed moderate reliability. Based on the most reliable indicators, a climate-sensitive Arid-Hot Small Ruminant Welfare Index (ASR-WI) was developed by weighting four welfare domains—Behaviour and Mental State, Environment, Nutrition, and Health. The findings confirm that a simplified welfare assessment protocol can be reliably implemented under intensive hot-arid conditions when clear scoring criteria and structured assessor training are provided. The resulting protocol and index offer a practical foundation for routine welfare monitoring under intensive hot-arid conditions, as well as for policymaking and future longitudinal research.

## Simple Summary

Small ruminants such as sheep and goats are one of the essential parts of food production in many hot-arid regions, including the United Arab Emirates and countries in the Gulf region. However, production from small ruminants under extreme heat and intensive housing conditions can create challenges for their well-being. This study developed a practical and easy-to-use welfare assessment tool to help farmers, students, and advisors evaluate the welfare of sheep and goats in these environments. We first reviewed many possible indicators of welfare and selected those that are simple to observe, reliable, and relevant to desert climates. We then trained university students to use the tool and tested it on sheep and goats at a research farm. The results showed that most indicators could be scored consistently by different people, especially those related to behaviour and health. A few indicators, such as signs of heat stress or body condition, were more difficult to score consistently across assessors. Based on these findings, we created a welfare index that combines the most reliable measures into a single score. This tool can support better daily management, guide training and education, and help improve the overall welfare of animals kept on farms in hot, challenging environments.

## 1. Introduction

The development of reliable and practical animal welfare assessment protocols is essential for ensuring the ethical management of livestock. Evaluating animal well-being at the flock or individual level requires assessment toolkits tailored to different husbandry systems. For sheep and goats, the global shift towards more intensive production systems has increased welfare risks related to housing, stocking density, and management practices, making systematic on-farm welfare assessment essential [1,2]. Despite their importance, there remain relatively few standardized and validated welfare assessment protocols specifically developed for sheep and goats [3].

Most existing welfare assessment frameworks for small ruminants have been developed in Western countries under temperate climatic conditions, including frameworks such as AWIN and approaches conceptually derived from Welfare Quality^®^ principles. While these tools represent important advances in welfare science and can be applied across a range of production systems, much of their development and validation has focused on temperate environments and pasture-based or mixed systems, and they may therefore have limited sensitivity to welfare challenges specific to intensive production under hot-arid climatic conditions. In countries such as the United Arab Emirates, sheep and goat farming is predominantly characterized by intensive or semi-intensive systems, prolonged exposure to high ambient temperatures, limited nocturnal cooling, and high stocking densities. These conditions pose specific welfare challenges—particularly related to heat stress, hydration, behavioural restriction and health—that are not fully addressed by welfare protocols developed for cooler climates and different management contexts [4,5]. This research gap is noteworthy given that small ruminants play a major role in the agricultural economies of many developing countries, particularly in arid and semi-arid regions, which together account for more than 70% of global small ruminant production [6]. In several hot-arid regions, sheep and goat husbandry has increasingly shifted towards intensive and semi-intensive production systems over recent decades, driven by urbanization, land-use limitations, and climate variability [7]. Therefore, the present study specifically focuses on intensive systems, as they represent the dominant production context in the UAE and pose distinct welfare challenges that require context-adapted assessment approaches.

Accordingly, there is a clear need for welfare assessment tools that are both scientifically robust and practically applicable under real farm conditions, particularly in resource-limited settings where time, labour, and technical expertise may be constrained. Simplified and feasible protocols facilitate routine welfare monitoring and support effective implementation at the farm level [8]. Selecting appropriate welfare indicators requires a systematic and harmonized approach to ensure the generation of quantitative and comparable welfare data. Recent work conducted for the European Food Safety Authority (EFSA) highlights the current lack of quantitative, harmonized and validated welfare data for farmed animals [9]. The development and implementation of such protocols also align with evolving market expectations and regulatory frameworks, in which animal welfare considerations are increasingly central to sustainable livestock production systems [10]. Moreover, standardized welfare assessment tools—defined by harmonized indicators, uniform scoring criteria, and consistent application procedures—can support governmental authorities and professional advisors in initiating consistent welfare monitoring, thereby informing future research, policy development, and industry guidance [11,12]. The aim of this study was to develop and pilot-validate a streamlined welfare assessment protocol for intensive sheep and goat farms in the UAE, structured to encompass the four fundamental welfare domains: Nutrition, Behaviour and Mental State, Environment, and Health. The protocol prioritizes animal-based measures within each domain to ensure that the indicators selected are both practical to implement and reflective of welfare status under local production conditions, including hot-arid climate, intensive housing systems, feeding practices, and routine management constraints typical of UAE farms.

## 2. Materials and Methods

### 2.1. Study Design, Study Site and Husbandry Conditions

This study was designed as a cross-sectional pilot validation study aimed at developing and evaluating a welfare assessment protocol for intensive sheep and goat production systems under hot-arid climatic conditions. The study was conducted at Al Foah Research Farm, United Arab Emirates University (UAEU), an intensive small-ruminant facility located in Al Ain, UAE. The region is characterized by an extremely hot-arid desert climate, with summer ambient temperatures frequently exceeding 45–50 °C and pronounced diurnal heat load. Heat stress has been shown to adversely affect thermoregulation, behaviour, feed intake, and health in small ruminants, thereby directly compromising animal welfare [13]. Under intensive production systems, such effects may be further exacerbated due to higher stocking densities and limited opportunities for behavioural thermoregulation.

A total of 100 animals—46 sheep (Local Sand Breed) and 54 goats (Local Sand Goats)—were assessed. Animals were clinically stable and represented mixed-age, mixed-parity groups typical of the farm’s production system. The selected sample size was considered sufficient for this pilot, reliability-focused study, as the primary objective was to evaluate inter-observer agreement of welfare indicators rather than to estimate population-level prevalence. In line with previous inter- and intra-observer reliability studies [14,15,16] in animal welfare research, sample size selection was guided by practical feasibility and the need to ensure multiple independent observations per indicator, rather than formal power calculations, which are not routinely applied in reliability-focused studies.

Animals were housed in semi-open, roofed sheds with sand bedding and slatted concrete feeding alleys. Shade and mechanical ventilation were provided; however, thermal load remained substantial during afternoon hours. Feeding followed routine intensive management practices, with animals receiving a commercial pelleted concentrate formulated for small ruminants (crude protein 14–16%) twice daily, supplemented with Rhodes grass hay. Fresh water was continuously available via drinkers and cleaned daily. Preventive health care included routine vaccination against clostridial diseases, internal/external parasite control, and weekly veterinary checks.

**Figure 1:**
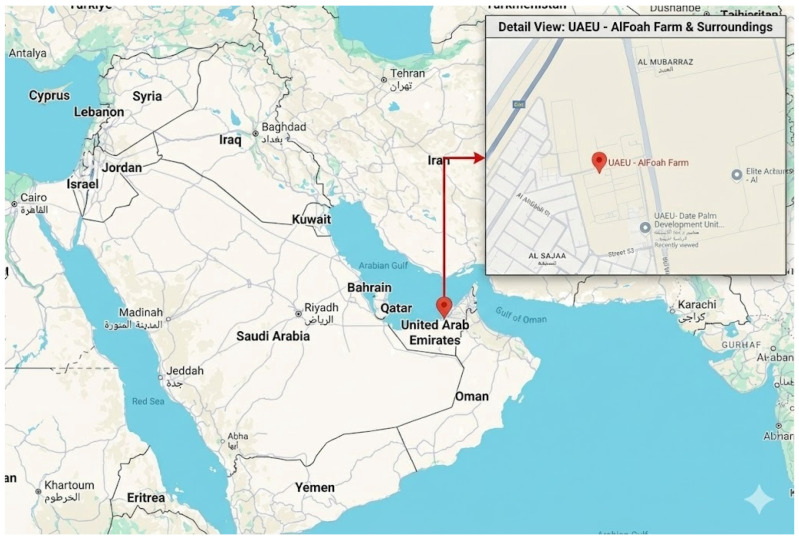
Location of the study site and study farm within the United Arab Emirates.

The geographical location of the study site within the United Arab Emirates is shown in Figure 1.

### 2.2. Screening of Candidate Indicators

To obtain a complete list of validated welfare indicators for small ruminants, the official supplementary dataset by de Jong et al. [17] was utilized. This dataset is publicly available on Zenodo (DOI: 10.5281/zenodo.10411335) and contains separate Excel tables for sheep and goats, including information on (i) assigned welfare domain (nutrition, environment, health, behaviour, mental state), (ii) indicator name and type, (iii) source and country of origin, (iv) animal category (age, sex, physiological status of animal, production purpose (dairy, meat or dual purpose)), (v) indicator definition and scoring criteria, and (vi) measurement context (on-farm or slaughter, manual or digital).

Species-specific datasets were subjected to targeted filtering aligned with the objectives of this study. Filters applied during data extraction and screening included (i) region and climate similarity, prioritizing studies conducted in hot, arid or semi-arid regions (e.g., GCC countries, North Africa, Australia, South Africa, Central Anatolia); (ii) production purpose, focusing on meat-oriented or dual-purpose systems where meat production predominates; (iii) production system, retaining indicators relevant to intensive and semi-intensive systems reflective of UAE farm structure; (iv) validation requirement, whereby only indicators validated within official welfare assessment protocols (e.g., AWIN Small Ruminants; welfare assessment frameworks developed following Welfare Quality^®^—Wageningen, The Netherlands, principles and widely applied in Europe), including documented inter or intra observer reliability testing and/or structured field application, or indicators repeatedly used in peer-reviewed studies were included. All indicators were categorized according to the Five Domains Model (nutrition, environment, health, behaviour, mental state) and by indicator type (animal-based, resource-based, and management- and environment-based indicators). The following were excluded: (i) studies performed in experimental or research farms as well as extensive or pastoral grazing systems; (ii) unvalidated indicators or anecdotal measures; and (iii) studies focusing solely on physiological stress biomarkers (e.g., cortisol) without accompanying behavioural or contextual measures. Pastoral and extensive systems were excluded because their management conditions differ substantially from the intensive and semi-intensive systems typical of the UAE, while experimental studies were excluded to prioritize indicators evaluated under commercial farm conditions.

**Figure 2:**
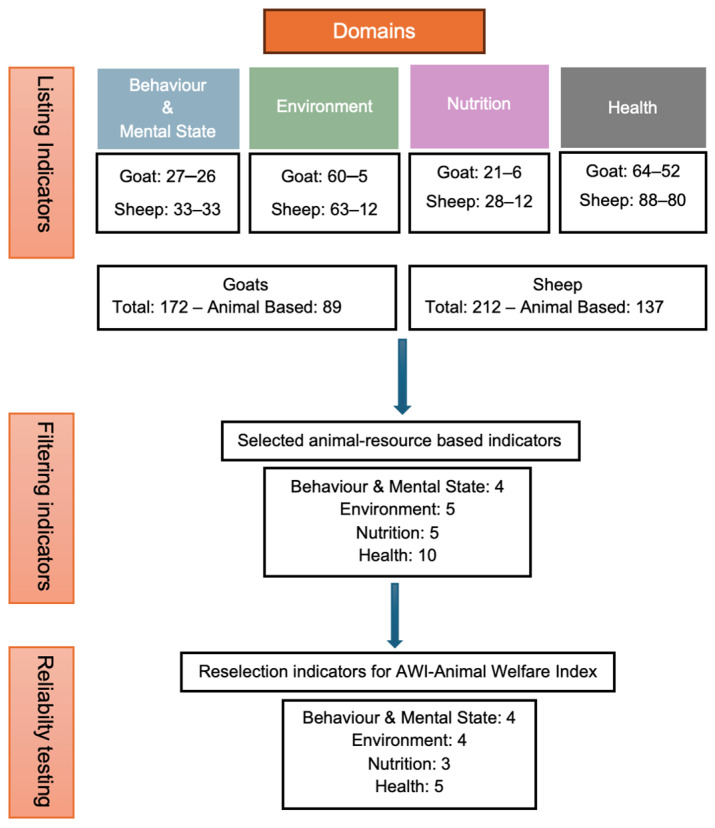
Summary of identification, screening, selecting, testing and finalization of welfare indicators included in the animal welfare index for welfare scoring in intensively managed sheep and goats in an arid-hot climate.

A summary of the identification, screening, selection, testing and finalization of welfare indicators included in the animal welfare index for intensively managed sheep and goats in arid-hot climates is presented in Figure 2. For each species and welfare domain, numerical ranges shown in the figure (e.g., Goat: 27–26 or Goat: 60–5) indicate the total number of candidate indicators initially identified from the literature, followed by the number of animal-based indicators retained after the initial screening and filtering process.

### 2.3. Development of the Assessment Protocol

Following finalization of the indicator list, four senior undergraduate students in Animal Science were each assigned to one of the four welfare domains—Behaviour and Mental State, Environment, Nutrition, and Health. Under the supervision and final approval of the animal welfare domain expert, the students translated the selected indicators into domain-specific scoring sheets, ensuring consistency with the refined indicator set and compatibility with intensive production conditions in the UAE. The scoring sheets followed a standardized format and were designed for practical field application. After expert review and revision, the final scoring sheets served as the operational basis of the on-farm assessment protocol. A complete description of all indicators, their operational definitions, scoring scales, and assessment procedures is provided in Supplementary Table S1 to ensure transparency and reproducibility. The senior students involved in the development of the scoring sheets did not participate in on-farm data collection or reliability scoring.

### 2.4. Assessor Training and On-Farm Application

Assessor training was conducted under the direct supervision of the animal welfare domain expert who oversaw protocol development.

Four senior undergraduate students delivered a structured 1.5 h training session to eight junior undergraduate students designated as field assessors. Training included visual and written explanations of each welfare indicator, supported by photographs and short video clips, explicit scoring criteria, and representative field examples aimed at standardizing interpretation across assessors.

Following training, the junior undergraduate assessors independently applied the protocol on-farm assessments at Al Foah Research Farm, evaluating 54 goats and 46 sheep. Each assessor completed the full assessment within approximately 1.5 h, following an identical observation sequence. This design facilitated evaluation of inter-observer consistency and assessment of the protocol’s practical feasibility under intensive hot-arid conditions. The same animal welfare domain expert supervised both the development of the assessment protocol and the assessor training process. The expert provided methodological guidance during indicator operationalization, reviewed and approved the domain-specific scoring sheets, and oversaw the training sessions and the subsequent on-farm application phase to ensure consistency, scientific accuracy, and adherence to the validated protocol framework. The animal welfare domain expert was also present during the on-farm application phase to provide real-time methodological oversight, without participating in the scoring process.

### 2.5. Reliability Assessment

Inter-rater reliability was evaluated to determine the consistency of scoring among the observers who independently assessed each welfare indicator. As multiple raters scored the same subjects using categorical scales, Fleiss’ Kappa (κ) was selected as the appropriate statistic for quantifying multi-rater agreement beyond chance [18]. Unlike Cohen’s Kappa—which is restricted to two raters—Fleiss’ Kappa accommodates varying numbers of raters per subject and is therefore suitable for the present dataset.

The reliability analysis followed the standard computational procedure. First, a rating matrix was constructed in which each row represented an assessed subject (e.g., individual animal, behavioural event, welfare indicator), and columns reflected the number of raters assigning each score category. Second, the proportions of ratings per category (p^−^) were computed across all subjects. Third, for each subject, per-subject agreement (P_i_) was calculated to quantify the extent to which raters converged on the same category more frequently than expected by chance. The overall observed agreement ($P^{-}$) and the expected agreement ($P_{e}^{-}$) were then obtained by averaging across subjects. Fleiss’ Kappa was computed as follows: κ values were interpreted according to the scale proposed by Landis and Koch [19], in which agreement ranges from poor (κ < 0.00) to slight (0.00–0.20), fair (0.21–0.40), moderate (0.41–0.60), substantial (0.61–0.80), and almost perfect agreement (0.81–1.00), providing a consistent framework for evaluating inter-observer reliability across all welfare indicators.

### 2.6. Development of Arid-Hot Small Ruminant Welfare Index (ASR-WI)

**Figure 3:**
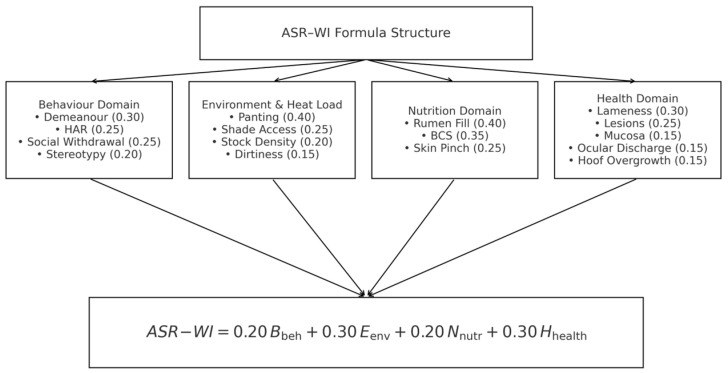
Weighted domain contributions to the ASR–WI and the resulting final index equation.

The Arid-Hot Small Ruminant Welfare Index (ASR-WI) was developed to provide a scientifically robust, reliability-based and climate-sensitive tool for evaluating the welfare of sheep and goats kept under hot desert conditions such as those in the United Arab Emirates. The index integrates the most reliable behavioural, environmental, nutritional, and health indicators identified in the dataset, prioritizing measures that are both highly repeatable and biologically relevant to arid-hot climates. All indicators were selected based on inter-observer reliability (Fleiss’ κ and percentage agreement) and refined using biological rationale derived from heat-stress physiology and welfare science, prioritizing indicators known to respond directly to thermal load, dehydration, altered feeding behaviour, and compromised health under hot-arid conditions. This integrated approach ensures that the index offers a comprehensive and accurate assessment tailored to the unique challenges faced by small ruminants in arid environments, enabling targeted interventions to improve animal well-being. Each domain score was normalized to a 0–100 scale by linearly rescaling the aggregated indicator scores within each domain, where higher values represent more favourable welfare indicators, and the final ASR-WI score was computed as a weighted sum of the four domains. This normalization allowed for an equitable comparison across domains, regardless of the initial number of indicators within each, and ensured that the aggregate score reflected a holistic welfare assessment. Formulas for each domain and the ASR-WI are given below and shown in Figure 3.

$$B\_beh=(\frac{0.30\cdot S_{D}+0.25\cdot S_{HAR}+0.25\cdot S_{SW}+0.20\cdot S_{St}}{2})\times 100$$

$$E\_env=(\frac{0.40\cdot S_{P}+0.25\cdot S_{Sh}+0.20\cdot S_{Sd}+0.15\cdot S_{Dir}}{2}\;)\times 100$$

$$N\_nutr=(\frac{0.40\cdot S_{Ru}+0.35\cdot S_{BCS}+0.25\cdot S_{Sk}}{2})\times 100$$

$$H\_health=(\frac{0.30\cdot S_{L}+0.25\cdot S_{Le}+0.15\cdot S_{Mu}+0.15\cdot S_{Oc}+0.15\cdot S_{Ho}}{2}\;)\times 100$$

ASR−WI=0.20·B_beh+0.30·E_env+0.20·N_nutr+0.30·H_health

Indicator inclusion in the ASR-WI followed a two-step decision process. First, indicators were required to meet a minimum level of inter-observer reliability (Fleiss’ κ ≥ 0.35, corresponding to at least moderate agreement). Indicators below this threshold were excluded regardless of their potential biological relevance. Second, indicators meeting the reliability criterion were evaluated for their biological relevance to welfare outcomes under hot-arid and intensive production conditions, informed by heat-stress physiology, animal welfare science, and expert field experience. While some indicators (e.g., panting) are directly associated with thermal load, others (e.g., hoof overgrowth) were retained due to their indirect but well-established links with welfare impairment in hot-arid intensive systems, where flooring conditions, reduced movement, and heat stress may exacerbate lameness and chronic discomfort.

Domain weights were determined through expert consensus involving two animal welfare scientists and three small ruminant production specialists with experience in intensive systems under hot-arid conditions. Consensus was reached through iterative discussion during protocol development, considering both the reliability of indicators within each domain and their biological importance for welfare outcomes under heat stress. For this pilot index, biological relevance was prioritized once minimum reliability criteria were satisfied, and the limitations of this expert-based approach are acknowledged.

## 3. Results

### 3.1. Inter-Observer Agreement of Welfare Indicators for Sheep and Goats

A comprehensive overview of the agreement levels for all assessed welfare indicators for both sheep and goats is presented in Table 1, categorized by their respective kappa values. For each indicator, the table details the calculated kappa statistic, a widely used measure of inter-rater agreement that accounts for chance agreement. The range and distribution of kappa values elucidate the varying degrees of inter-observer reliability across the diverse set of welfare parameters, offering critical insights into the consistency and reliability of the developed assessment protocol.

### 3.2. Behaviour and Mental State

The Behaviour and Mental State domain showed consistently high inter-observer reliability across both sheep and goat assessments. Fleiss’ Kappa values (κ ≈ 0.80), together with an overall agreement of approximately 91%, indicate strong concordance among the eight trained assessors. Behavioural indicators such as demeanour, social withdrawal, and human–animal relationship were clearly defined and easily identifiable during field observations, which contributed to the low variability between observers. Overall, this domain provided the most stable behavioural evaluations, suggesting that the combination of structured training and well-defined operational criteria facilitated a high level of inter-observer scoring. Nevertheless, minor inconsistencies may still occur in the assessment of subtle or low-intensity behaviours, such as mild stereotypies, which are inherently more difficult to detect with uniform precision.

### 3.3. Environment

The Environment domain demonstrated the lowest inter-observer reliability among the four welfare categories, with Fleiss’ Kappa values of approximately κ = 0.65 for sheep and κ = 0.63 for goats and agreement rates ranging between 82 and 85%. The observed variation was likely driven by context-dependent or inherently subjective indicators, such as panting related to thermal stress or the condition of equipment, where assessors may have differed in their interpretation of severity or scoring thresholds. Overall, reliability in this domain can be considered moderate, indicating the need for clearer scoring criteria, enriched visual examples, and more frequent calibration exercises to improve consistency.

Indicators requiring more straightforward visual assessment, such as access to shade/shelter and stocking density, showed good inter-observer agreement across assessors, whereas indicators involving greater evaluator judgement—such as panting score related to thermal stress and condition of equipment—contributed to higher variability in scoring. Enhancing visual reference materials and refining threshold definitions would likely strengthen future reliability outcomes in this domain.

### 3.4. Nutrition

The Nutrition domain demonstrated good inter-observer reliability across both species, with Fleiss’ Kappa values ranging from κ ≈ 0.70 to 0.72 and agreement levels of approximately 84%. Most variability originated from tactile or semi-quantitative indicators, such as body condition score (BCS) and the skin-pinch hydration test, both of which require subjective judgement and direct physical interaction with the animal. Despite this, overall reliability remained solid, indicating that nutritional welfare can be assessed consistently when assessors receive standardized training. Visual indicators were scored with notable stability, whereas handling-based measures showed greater observer sensitivity, highlighting the need for continued calibration using shared reference materials and harmonized scoring guides.

### 3.5. Health

The health domain achieved the highest reliability scores among all categories, with Fleiss’ Kappa values of approximately κ = 0.83 and overall agreement levels near 91%. Indicators such as lameness, ocular discharge, and mucosal colour were highly objective, visually distinct, and clinically recognizable, resulting in excellent consistency across observers. Both sheep and goats demonstrated parallel reliability patterns, underscoring the high reliability and cross-species applicability of these clinical welfare measures. Overall, the Health domain exhibited very high reproducibility, confirming that trained assessors can evaluate physical health indicators with strong uniformity. Minor variability may still occur in subclinical or subtle presentations, although such cases are inherently more difficult to score consistently.

**Figure 4:**
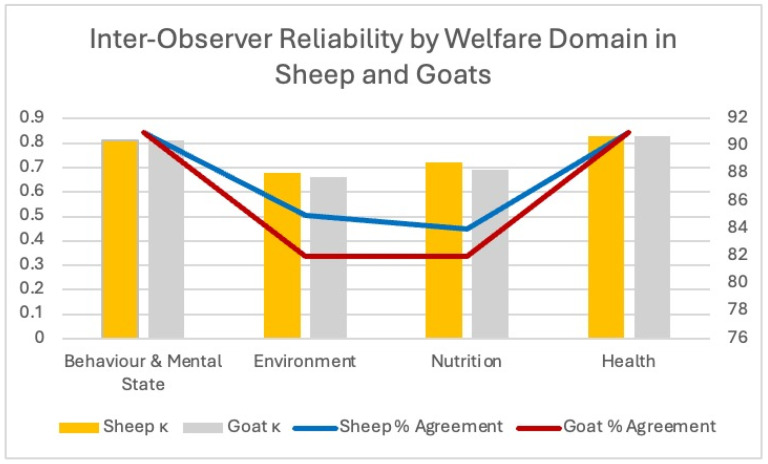
Inter-observer reliability (κ and % agreement) across the four welfare domains in sheep and goats.

As illustrated in Figure 4, inter-observer reliability was generally high across all welfare domains for both species. Behaviour and Mental State showed strong agreement, with κ values approaching 0.80 in sheep and 0.75 in goats, reflecting good consistency in behavioural scoring. Agreement was lowest in the Environment domain for both species (κ ≈ 0.45–0.50), indicating greater subjectivity in environmental cleanliness and shade-access evaluations. Nutrition indicators demonstrated moderate reliability (κ ≈ 0.55 in sheep and 0.60 in goats), while the Health domain exhibited the highest reliability overall, with κ values exceeding 0.80 and percentage agreement reaching approximately 90% in both species. These results suggest that indicators requiring direct individual assessment—such as lameness, lesions, or mucosal colour—tend to yield more consistent scoring between observers compared with broader environmental assessments.

## 4. Discussion

The development of a simplified welfare assessment protocol for intensive sheep and goat farms in the United Arab Emirates is critical for improving animal husbandry practices and ensuring sustainable livestock production in the region [14]. In this study, a pilot reliability-focused approach was adopted to evaluate whether a streamlined protocol could be consistently applied under real farm conditions, addressing a key gap in welfare assessment tools adapted to intensive systems in arid climates. In the context of this study, the term “simplified” refers to a reduction in the number and complexity of indicators and assessment steps, with emphasis on indicators showing high inter-observer reliability and clear field applicability, rather than a reduction in scientific rigour.

The inter-observer reliability analysis revealed substantial to almost perfect agreement for the majority of indicators assessed across behavioural, health, and nutritional domains, particularly those related to resource-based measures and clearly definable animal-based indicators, such as lameness, ocular discharge, mucosal colour, and overt behavioural responses. These findings are in line with previous sheep and goat welfare studies reporting higher agreement for indicators with explicit operational definitions and observable outcomes [1,8,14]. In contrast, certain animal-based indicators, such as body condition score, exhibited lower agreement, suggesting a need for more detailed scoring criteria or enhanced assessor training. This observation is consistent with earlier research highlighting persistent challenges in achieving consistent scoring for subjective indicators, even among trained observers [8,15]. For instance, previous studies evaluating animal-based indicators in sheep and goat welfare have similarly identified lower agreement levels for indicators such as body condition score, claw health, and skin or callus-related measures, particularly when scoring relies on visual or tactile judgment [8]. Previous studies on sheep and goat welfare assessment have highlighted the importance of selecting indicators that are both biologically meaningful and feasible for on-farm application [20,21]. Reviews and field-based studies have shown that animal-based indicators such as body condition, lameness, and behavioural responses are central to welfare evaluation, but their reliability may vary depending on observer training and assessment context [22,23,24,25]. Similar challenges have been reported in welfare assessment protocols developed for small ruminants across different production systems, reinforcing the need for context-specific adaptation of indicators [17,22,23,24,25].

The variability in agreement observed for these indicators underscores the importance of incorporating inter-observer reliability testing as a core component of welfare protocol development, particularly when qualitative or semi-quantitative indicators are included [16]. A key contribution of the present study lies in its explicit focus on inter-observer reliability as a prerequisite for practical welfare assessment. Previous research has demonstrated that even well-established welfare indicators may show variable agreement when applied under field conditions, particularly for qualitative or semi-quantitative measures [15,23]. By prioritizing indicators with acceptable reliability levels and supporting their application through structured training and standardized scoring sheets, the current protocol addresses a critical requirement for routine on-farm welfare monitoring [21,24].

The present findings confirm that, with appropriate training and standardized scoring sheets, reliable welfare assessments are achievable in intensive sheep and goat farms operating under hot-arid conditions. At the same time, the results highlight the need for continued refinement of specific indicators and calibration procedures to minimize observer-related variability, as also reported in comparable welfare assessment studies [1]. Integrating objective measures, where feasible, including digital tools or precision livestock farming approaches, may further enhance consistency and reduce reliance on subjective interpretation.

Most existing small ruminant welfare assessment protocols have been developed under temperate climatic conditions and in pasture-based or mixed production systems [17]. In contrast, intensive production systems in hot-arid environments pose specific welfare challenges related to heat stress, hydration, and restricted behavioural expression [2,26]. The present findings highlight the importance of adapting welfare assessment tools to these conditions, ensuring that selected indicators remain relevant and interpretable under high thermal load. The significance of this study lies in its focus on intensive production systems in a hot-arid environment, a context that remains underrepresented in existing small ruminant welfare protocols, which have largely been developed for temperate regions. By prioritizing reliable animal-based indicators while retaining essential environment-related measures, the proposed protocol offers a context-specific and practically applicable framework for welfare assessment in the United Arab Emirates and similar regions.

Several limitations of this pilot study should be acknowledged. The protocol was applied on a single intensive farm, and the primary focus was on inter-observer reliability rather than on other validation aspects such as sensitivity or construct validity. These limitations are typical of early-stage protocol development studies and highlight the need for future multi-farm applications and broader validation efforts to further strengthen the robustness and applicability of the proposed welfare assessment framework.

## 5. Conclusions

In conclusion, this pilot study demonstrated that a simplified and structured welfare assessment protocol can be reliably applied by trained assessors following standardized scoring sheets and a structured training procedure in intensive sheep and goat farms operating under hot-arid conditions. High inter-observer agreement was achieved across key welfare domains, particularly for health- and behaviour-related indicators, confirming the feasibility of using clearly defined animal-based measures in this context. Lower agreement observed for some tactile or semi-quantitative indicators highlights the need for continued refinement of scoring criteria and calibration procedures. Overall, the proposed protocol provides a practical basis for routine welfare monitoring in intensive systems in arid regions and represents an important first step toward broader validation and implementation across diverse production settings.

## Figures and Tables

**Table 1:** Inter-Observer Reliability of Welfare Indicators Across All Domains (Behaviour, Nutrition, Health, Housing) in Goats and Sheep (κ/% Agreement/Reliability).

Domain	Indicators	Species (κ/% Agreement/Reliability)	
		Goats	Sheep
Behaviour and Mental State	Demeanour	κ = 1.00 (100%; Very High)	κ = 1.00 (100%; Very High)
	Human–Animal Relationship	κ = 1.00 (100%; Very High)	κ = 0.70 (87.5%; Good)
	Social Withdrawal	κ = 0.35 (62%; Moderate)	κ = 1.00 (100%; Very High)
	Stereotypic Behaviour	κ = 0.65 (88%; Good)	κ = 0.55 (75%; Moderate)
Environment	Access to Shade/Shelter	κ = 0.65 (85%; Good)	κ = 0.70 (88%; Good)
	Condition of Equipment	κ = 0.55 (75%; Moderate)	κ = 0.60 (80%; Good)
	Dirtiness (Fecal Soiling)	κ = 0.70 (88%; Good)	κ = 0.75 (88%; Good)
	Panting (Thermal Stress)	κ = 0.40 (65%; Moderate)	κ = 0.45 (68%; Moderate)
	Stocking Density	κ = 1.00 (100%; Very High)	κ = 0.90 (95%; Very High)
Nutrition	Cleanliness of Drinking Area	κ = 0.80 (90%; Good)	κ = 0.80 (90%; Good)
	Cleanliness of Feeding Area	κ = 0.65 (85%; Good)	κ = 0.70 (85%; Good)
	Rumen Fill	κ = 1.00 (100%; Very High)	κ = 1.00 (100%; Very High)
	Body Condition Score (BCS)	κ = 0.55 (70%; Moderate)	κ = 0.60 (75%; Good)
	Skin Pinch Test (Hydration)	κ = 0.45 (68%; Moderate)	κ = 0.45 (68%; Moderate)
Health	Body and Head Lesions	κ = 1.00 (100%; Very High)	κ = 1.00 (100%; Very High)
	Excessive Itching	κ = 1.00 (100%; Very High)	κ = 0.85 (93%; Very High)
	Fecal Soiling	κ = 0.65 (80%; Good)	κ = 0.65 (80%; Good)
	Fleece Loss and Quality	κ = 0.55 (70%; Moderate)	κ = 0.55 (70%; Moderate)
	Hoof Overgrowth	κ = 0.75 (88%; Good)	κ = 1.00 (100%; Very High)
	Lameness	κ = 0.90 (95%; Very High)	κ = 0.90 (95%; Very High)
	Mastitis-Udder Lesions	κ = 0.55 (70%; Moderate)	κ = 0.55 (70%; Moderate)
	Mucosa Colour	κ = 1.00 (100%; Very High)	κ = 1.00 (100%; Very High)
	Ocular Discharge	κ = 1.00 (100%; Very High)	κ = 1.00 (100%; Very High)
	Respiratory Quality	κ = 0.45 (68%; Moderate)	κ = 0.45 (68%; Moderate)

## Data Availability

The data supporting the reported results are available on request from the corresponding author. The data are not publicly available due to privacy and ethical restrictions.

## Supplementary Materials

The following supporting information can be downloaded at: https://www.mdpi.com/article/10.3390/ani16040563/s1, Table S1: Scoring Sheets Used in the On-Farm Welfare Assessment Protocol.

## Abbreviations

The following abbreviations are used in this manuscript:

ASR-WI	Arid-Hot Small Ruminant Welfare Index
AWIN	Animal Welfare Indicators
BCS	Body Condition Score
EFSA	European Food Safety Authority
κ	Fleiss’ Kappa Agreement Statistic
PLF	Precision Livestock Farming
