# Supplementary material for: Developing a Practical Welfare Assessment Tool for Intensive Sheep and Goat Farming in Hot-Arid Regions: Pilot Validation in the United Arab Emirates
Source: Animals (Basel). 2026 Feb 11;16(4):563. doi: 10.3390/ani16040563 (PMC12937362; doi:10.3390/ani16040563)
Supplement: Supplementary file 1 [file animals-16-00563-s001.zip › Table S1.pdf]

UAEU-SHEEP&GOATS WELFARE ASSESSMENT FORM

Farm: _________ **Date:** _________ **Assessor:** _________ **Sub-group:** Indoor / Semi-indoor / Outdoor

DOMAIN 1- BEHAVIOUR AND MENTAL STATE

Indicator	Method / Observation Guide	No. of Animals / Observation	Scoring Criteria	Score (✓)	Comments / Evidence
**1. Demeanour**	Observe from ~2 m without disturbing. Assess posture, head carriage, responsiveness, and separation from flock.	≥ 20 ewes per sub-group	0 = Dull – Depressed – Unresponsive<br>1 = Bright – Alert – Responsive	☐ 0 ☐ 1	
**2. Human–Animal Relationship**	Familiar stockperson approaches flock as in daily inspection; assessor observes reactions from a distance.	≥ 20 ewes per sub-group	2 = Majority approach / calm (<10 % flight)<br>1 = Mixed reaction (10–30 % flight)<br>0 = Avoid / panic (>30 % flight)	☐ 0 ☐ 1 ☐ 2	
**3. Social Withdrawal**	Observe flock undisturbed for 20 min. Count animals separated, inactive, or not grooming / feeding.	≥ 20 ewes per sub-group	2 = ≥ 85 % together → Good integration<br>1 = 70–85 % together → Slight concern<br>0 = < 70 % together → Welfare issue	☐ 0 ☐ 1 ☐ 2	
**4. Stereotypic Behaviour**	Observe 10 % of flock (~20 sheep) individually during non-feeding period. Record repetitive pacing, star-gazing, wool-pulling / biting.	~ 20 sheep (only housed flocks)	2 = None observed<br>1 = Occasional (<5 %)<br>0 = Frequent (>5 %)	☐ 0 ☐ 1 ☐ 2	

DOMAIN 2- ENVIROMENT

Indicator	Method / Observation Guide	Observation Unit / No. of Animals	Scoring Criteria	Score (✓)	Comments / Evidence
**1. Access to Shade / Shelter**	Record % of flock with proper access at time of visit. Observe overall area under UAE conditions (midday hours).	Entire flock area	0 = No access<br>1 = Partial (≈50 %)<br>2 = Full (≈100 %)	☐ 0 ☐ 1 ☐ 2	
**2. Condition of Equipment**	Inspect feeders, drinkers, fences, and handling facilities for cleanliness, functionality, and safety.	All equipment used by flock	0 = Poor/Unsafe – broken, dirty, risky<br>1 = Good/Safe – clean, intact, functional	☐ 0 ☐ 1	
**3. Dirtiness (Faecal Soiling)**	Visually assess one side + hindquarters + belly. Check all if ≤ 50, otherwise 30 randomly.	30 sheep (if > 50 flock)	0 = Dirty<br>1 = Moderate<br>2= Clean	☐ 0 ☐ 1 ☐ 2	
**4. Panting (Thermal Stress)**	Observe 12:00–15:00 in shade. Count flank movements or open-mouth panting. Only unhandled animals.	≥ 20 ewes	0 = Severe (> 40 BPM / open mouth)<br>1 = Mild (30–40 BPM)<br>2 = Normal (< 30 BPM)	☐ 0 ☐ 1 ☐ 2	
**5. Stocking Density (Space per Sheep)**	Measure usable pen area (m^2) ÷ no. of sheep. Exclude feeders or obstacles.	Each pen	2 = Good (≥ 1.5 m^2 ewes / ≥ 2 m^2 ewes + lambs)<br>1 = Adequate (1–1.5 m^2 / 1.5–2 m^2)<br>0 = Poor (≤ 1 m^2 / < 1.5 m^2)	☐ 0 ☐ 1 ☐ 2	

DOMAIN 3- NUTRITION

Indicator	Method / Observation Guide	No. of Animals / Observation	Scoring Criteria	Score (✓)	Comments / Evidence
**1. Cleanliness of Drinking Area**	Visual inspection of troughs for feed residues, feces, or algae contamination.	Per pen or per farm	1 = Clean (no contamination)<br>0.5 = Moderate<br>0 = Dirty (feces / algae)	☐ 0 ☐ 0.5 ☐ 1	
**2. Cleanliness of Feeding Area**	Inspect feeding area and surroundings for feces, spoiled feed, or mud.	Per pen or per farm	1 = Clean<br>0.5 = Moderate<br>0 = Dirty	☐ 0 ☐ 0.5 ☐ 1	
**3. Rumen Fill**	Observe left flank (between hip bone and ribs).	≥ 20 ewes	0 = Deeply sunken → welfare concern<br>1 = Normal / convex	☐ 0 ☐ 1	
**4. Body Condition Score (BCS)**	Body condition is assessed by palpation of the spine in the lumbar region just after the last rib. For welfare purposes animals are considered thin if they score below 2.0 on this scale, emaciated if they are ≤ 1.0, and fat if they are > 4.0. This system is used for all sheep breeds and all purposes of use (Russell et al., 1969).	≥ 20 ewes (per flock)	0 = Emaciated (≤ 1.0)<br>1 = Thin (< 2.0)<br>2 = Good (2–< 4.0)<br>3= Fat (> 4.0)	☐ 0 ☐ 1 ☐ 2 ☐ 3	
**5. Skin Pinch Test (Hydration)**	Pinch a small fold of skin on the neck/shoulder and note the time for it to return.	≥ 10 ewes	2= < 2 s -Normal<br>1=2–3 s-Mild dehydration<br>0= > 4 s -Severe dehydration	☐ 0 ☐ 1 ☐ 2	

DOMAIN 4- HEALTH

Indicator	Method / Observation Guide	No. of Animals / Observation	Scoring Criteria	Score (✓)	Comments / Evidence
**1. Body and Head Lesions**	Handle sheep in race or pen. Inspect head, ears, face, and body for recent or healed lesions.	≥ 20 animals or 10 % of flock	0 = Major (≥ 1 large > 1×2 cm or > 4 cm linear, inflamed or swollen area)<br>1 = Minor (1–2 small < 1×2 cm or < 4 cm linear<br>2 = No lesions	☐ 0 ☐ 1 ☐ 2	
**2. Excessive Itching**	Observe flock undisturbed 20 min; record rubbing or scratching lasting ≥ 5 min.	Whole flock	2 = None<br>1 = Few (< 10 %)<br>0 = Many (≥ 10 %)	☐ 0 ☐ 1 ☐ 2	
**3. Faecal Soiling**	Examine hindquarters.	≥ 20 animals or 10 %	1= Clean<br>0= Soiled	☐ 0 ☐ 1	
**4. Fleece Loss & Quality**	Observe wool for bald/loose patches; confirm by handling if needed.	≥ 20 animals or 10 %	2 = No loss<br>1 = Mild<br>0 = Severe	☐ 0 ☐ 1 ☐ 2	
**5. Hoof Overgrowth**	Restrain on hard surface; inspect hooves for length/shape.	≥ 20 animals or 10 %	1 = Normal<br>0 = Overgrown	☐ 0 ☐ 1	
**6. Lameness**	Observe at rest and walking; confirm individually in race.	Whole flock (≥ 20 for check)	2 = None<br>1 = Mild (1–5 %)<br>0 = Severe (> 5 %)	☐ 0 ☐ 1 ☐ 2	
**7. Mastitis / Udder Lesions**	Handle lactating ewes; inspect & palpate udder for lumps/hardness/asymmetry.	≥ 20 ewes or 10 %	1 = Absent<br>0 = Present	☐ 0 ☐ 1	
**8. Mucosa Colour**	Gently pull lower eyelid; inspect conjunctiva.	≥ 20 ewes or 10 %	2 = Pink (normal)<br>1 = Pale<br>0 = Very pale	☐ 0 ☐ 1 ☐ 2	
**9. Ocular Discharge**	While restrained, check eyes for discharge.	≥ 20 ewes or 10 %	1 = Absent<br>0 = Present	☐ 0 ☐ 1	
**10. Respiratory Quality**	Observe/listen for coughing or nasal discharge while restrained.	≥ 20 ewes or 10 %	2 = Normal<br>1 = Audible / Nasal<br>0 = Coughing	☐ 0 ☐ 1 ☐ 2	
